# Broadly neutralizing single-domain antibody targeting the spike NTD displays therapeutic efficacy against diverse SARS-CoV-2 variants

**DOI:** 10.3389/fimmu.2026.1799333

**Published:** 2026-03-23

**Authors:** Anastasia I. Korobkova, Irina A. Favorskaya, Inna V. Shuliakova, Anna A. Iliukhina, Daria M. Grousova, Irina A. Alekseeva, Ekaterina I. Ryabova, Artem A. Derkaev, Valentin V. Azizyan, Ilya D. Zorkov, Alina S. Dzharullaeva, Amir I. Tukhvatulin, Ilias B. Esmagambetov, Vladimir V. Prokofiev, Daria V. Voronina, Olga V. Zubkova, Dmitry V. Shcheblyakov, Denis Y. Logunov

**Affiliations:** 1 Department of Genetics and Molecular Biology of Bacteria, Federal State Budget Institution “National Research Centre for Epidemiology and Microbiology Named after Honorary Academician N.F. Gamaleya” of the Ministry of Health of the Russian Federation, Moscow, Russia; 2 Bacterial Infection Department, Federal State Budget Institution “National Research Centre for Epidemiology and Microbiology Named after Honorary Academician N.F. Gamaleya” of the Ministry of Health of the Russian Federation, Moscow, Russia; 3 Department of the National Virus Collection, Federal State Budget Institution “National Research Centre for Epidemiology and Microbiology Named after Honorary Academician N.F. Gamaleya” of the Ministry of Health of the Russian Federation, Moscow, Russia; 4 Medical Microbiology Department, Federal State Budget Institution “National Research Center for Epidemiology and Microbiology Named after Honorary Academician N.F. Gamaleya” of the Ministry of Health of the Russian Federation, Moscow, Russia; 5 Department of Immunology, Federal State Budget Institution “National Research Centre for Epidemiology and Microbiology Named after Honorary Academician N.F. Gamaleya” of the Ministry of Health of the Russian Federation, Moscow, Russia

**Keywords:** broadly neutralizing antibodies, COVID-19, SARS-CoV-2, sdAbs, single-domain antibodies, therapeutic efficacy

## Abstract

The continuous evolution of SARS-CoV-2 virus has led to the emergence of multiple Omicron descendants resistant to previously approved therapeutic antibodies. Therefore, cross-reactive antibodies to conserved neutralizing epitopes are urgently needed to protect immunocompromised patients and individuals at high risk of severe COVID-19. Here we report the characterization of 1p2B5, a single-domain antibody targeting N-terminal domain (NTD) of SARS-CoV-2 S protein. 1p2B5 exerts broad neutralizing activity against diverse SARS-CoV-2 variants. The Fc-fused form of the antibody demonstrates enhanced neutralizing potency *in vitro*, inhibiting both the earliest and currently circulating subvariants with nanomolar activity. Systemic therapeutic administration of 1p2B5-Fc at a dose of 1 mg/kg protects Syrian hamsters and hACE2-transgenic mice from challenge with evolutionarily distant SARS-CoV-2 variants. Our findings highlight the potential of NTD-specific monoclonal antibodies for prophylaxis and therapeutic intervention.

## 1 Introduction

The COVID-19 pandemic caused by SARS-CoV-2 virus in 2020 has led to 7 million deaths and has demonstrated the critical need for effective prophylaxis and therapy (1). The long-term circulation of the virus in the human population has given rise to a wide variety of SARS-CoV-2 variants capable of inducing disease outbreaks. Since 2022, Omicron subvariants have spread rapidly and have surpassed predominant variants. The latest Omicron sublineages have accumulated multiple mutations that allow the virus to evade immune responses induced by vaccination or previous infection and reduced the efficacy of therapeutic antibodies (2).

The most crucial SARS-CoV-2 mutations are accumulated in the spike (S) glycoprotein that is essential for viral entry into host cells. The spike protein is located on the surface of the viral envelope and binds to the human angiotensin-converting enzyme 2 (ACE2) receptor, mediating virus attachment and internalization (3). Most neutralizing antibodies produced by the host’s immune system target SARS-CoV-2 S glycoprotein. Potent neutralizing antibodies predominantly recognize S1 subunit of S protein including the receptor-binding domain (RBD) and the N-terminal domain (NTD). The RBD plays a key role in SARS-CoV-2 binding to the ACE2 receptor. Therefore, many RBD-directed antibodies (Abs) neutralize the virus by blocking the RBD-ACE2 interaction. However, only a few of them bind to conserved epitopes, remaining effective against emerging SARS-CoV-2 variants. Consequently, the identification of conserved neutralizing epitopes located beyond the RBD is the important goal for therapeutic agents development (4).

The N-terminal domain of S protein is involved in viral entry process, likely by regulating conformational changes in the S1 subunit (4). The first published anti-NTD neutralizing antibodies targeted so-called supersite, the most immunogenic region in the domain. The majority of these antibodies lost activity against Omicron subvariants (5). Later, neutralizing antibodies targeting conserved NTD epitopes outside the supersite were identified (4, 6, 7). However, their neutralizing activity is often lower than that of RBD-specific antibodies (1, 8). Neutralization of the virus by anti-NTD antibodies can be achieved through several mechanisms. Some antibodies can block viral attachment by indirect inhibiting the interaction between the S protein and the ACE2 (9, 10). Other Abs are capable of blocking later stages after ACE2 engagement (11–13). However, for some Abs, mechanisms of neutralizing activity have not been fully understood (11).

Here, we present the NTD-targeted single-domain antibody 1p2B5 with potent neutralizing activity against SARS-CoV-2. Despite the NTD has accumulated multiple mutations, 1p2B5 is able to neutralize various SARS-CoV-2 variants, including the earliest and the recently emerged ones, at nanomolar concentrations. The Fc-fused form of 1p2B5 antibody displays improved neutralizing activity *in vitro* and therapeutic efficacy *in vivo*. Systemic administration of 1p2B5-Fc at dose of 1 mg/kg protects animals from challenge with different SARS-CoV-2 variants. The remarkable protective efficacy of 1p2B5-Fc underscores its potential for clinical application.

## 2 Materials and methods

### 2.1 Cell lines, bacterial strains

Vero E6 and HEK293 cells were obtained from Russian collection of vertebrate cell lines. Vero E6 cell line was used to perform live SARS-CoV-2 virus neutralization assay and membrane fusion assay. HEK293 cells were used in ADCC and ADCP assays. The measurements of the ADCC and ADCP activities of antibodies were performed using Jurkat-Lucia™ NFAT-CD16 and Jurkat-Lucia™ NFAT-CD32 reporter cell lines (Invivogen), respectively. 1p2B5-Fc antibody was expressed in CHO-S cells (Thermo Fisher Scientific, USA).

*E. coli* strain Rosetta (DE3) (Merck, USA) was used for the 1p2B5 single-domain antibody production. *E. coli* strain DH5α (Thermo Fisher Scientific, USA) was used for cloning applications.

### 2.2 Viruses

SARS-CoV-2 virus variants were obtained from the State Collection of Viruses (Gamaleya Research Center, Moscow, Russia): Wuhan B.1.1.1 (hCoV-19/Russia/Moscow_PMVL-1/2020), Omicron XBB 1.9.1 (hCoV-19/Russia/MOW-PMVL-OM0223O11/2023), Omicron 1.9.1.5.1 Eris EG.5.1 (hCoV-19/Russia/SPE-RII-21139S/2023), Omicron XBB 1.16 (hCoV-19/Russia/MOW-PMVL-OM0223O420/2023), Omicron B.1.1.529 JN.1 (hCoV-19/Russia/MOW-PMVL-LSCV-LD134/2023), Omicron B.1.1.529 KS.1 (hCoV-19/Russia/SPE-RII-MH183935S/2024), XFG.3.

### 2.3 Single-domain antibody production and purification

The 1p2B5 single-domain antibody was produced in *E. coli* strain Rosetta (DE3) (Merck, USA). A plasmid vector pET28b, encoding 1p2B5 fused to a PelB signal sequence, Myc-tag and 6xHis-tag, was transformed into the bacterial cells. A single transformed colony was inoculated into 2xYT medium supplemented with 100 μg/mL kanamycin. The culture was grown at 37 °C with shaking until OD600 reached 0.6. The VHH production was induced by adding 0.1 mM Isopropyl β-D-1-thiogalactopyranoside (IPTG), followed by overnight incubation at 30 °C with shaking.

The next day, bacterial cells were harvested and lysed by two freeze-thaw cycles. The cell pellet was resuspended in phosphate buffer saline (PBS) (50 mM Na_2_HPO_4_ + NaH_2_PO_4_, 0.3 M NaCl, pH = 7.4) containing lysozyme (0.2 mg/mL), PMSF (1 mM), MgCl_2_ (1 mM) and benzonase (50 unit/mL) and incubated with gentle shaking for 30 minutes at room temperature. The resulting cell lysate was centrifuged (5000 g, 20 min, 4 °C), and the supernatant was loaded onto HisTrap column (Cytiva, USA) packed with IMAC Sepharose 6 Fast Flow resin (Cytiva, USA), pre-equilibrated with PBS. The column was washed with 30 mM imidazole in PBS, and then the VHH was eluted with 300 mM imidazole in PBS.

### 2.4 ELISA

The binding activity of antibodies was examined by the indirect ELISA. Briefly, 96-well ELISA plate (Costar, USA) was coated overnight at 4 °C with 100 ng per well of the recombinant SARS-CoV-2 S-protein (Delta, Omicron B.1.1.529 or XBB.1 variant). After washing with PBS containing 0.05% Tween-20 (TPBS), wells were blocked with 5% (w/v) skimmed milk in TPBS. Serial dilutions of 1p2B5 or 1p2B5-Fc were added to the wells. After incubation at 37 °C for 1 h, the wells were washed as above, and HRP-conjugated anti-Myc-tag antibodies (Abcam, UK, ab1326) or anti-human-IgG antibodies (Sigma-Aldrich, USA, A8667) were added for 1 h at 37 °C. Then, the wells were rinsed with TPBS and TMB substrate solution (Imtek, Russia) was added. After 15-minute incubation in the dark, the reaction was stopped with 1 M H_2_SO_4_. The absorbance at 450 nm (OD450) was measured using a Multiskan FC (Thermo Fisher Scientific, USA).

A competitive ELISA was performed to evaluate the ability of 1p2B5 antibody to inhibit the binding of SARS-CoV-2 S protein (XBB.1 variant) to the hACE2 receptor. Dilutions of the VHH 1p2B5 were pre-incubated with biotin-labeled S protein for 30 minutes at 37 °C. The S protein was biotinylated using an EZ-Link Sulfo-NHS-LC-Biotinylation kit (Thermo Fisher Scientific, USA). The mixtures were added to the plate coated with recombinant hACE2 protein (400 ng of hACE2 per well). Bound S protein was detected using HRP-conjugated Streptavidin (Thermo Fisher Scientific, #21140). The subsequent TMB substrate reaction and stop steps were performed as described above. A competing camel immune serum was used as positive control, while VHH, which does not bind to the S protein, was used as negative control of signal inhibition.

Inhibition of S protein binding to the hACE2 receptor was calculated using the formula: [(signal in the well without antibody – signal in the well with antibody)/signal in the well without antibody] x 100%.

### 2.5 Cloning, expression and purification of Fc-fused forms of 1p2B5

The 1p2B5-Fc coding sequence was generated by fusing the sequences of the 1p2B5 single-domain antibody and Fc fragment of human IgG1 by overlap-extension PCR. For secretion of the recombinant protein, the IgG1 signal peptide (MGWSLILLFLVAVATRVLS) was added to the N-terminus. Obtained sequence was cloned into pCEP4 mammalian expression vector. The antibody was expressed in CHO-S cell line via transient transfection with recombinant pCEP4 vector. For transfection, CHO-S cells were resuspended at a density of 1x10^6^ cells/ml in EmCD CHO-S 203 Medium (Eminence, China) and plasmid DNA (1 µg per 10^6^ cells) premixed with polyethylenimine 2500 (PEI 25K) transfection reagent (Polyscience, USA) was added. The cells were incubated overnight at 37 °C and 5% CO_2_, two-fold diluted in EmCD CHO^®^121 Basal Medium (Eminence, China), and then incubated for 10–14 days under the same conditions. Starting from 3 days after transfection, the cells were supplemented with EmCD CHO^®^121 Feed A (Eminence, China) и EmCD CHO^®^121 Feed B (Eminence, China). The 1p2B5-Fc antibody was purified from the culture supernatant using protein A affinity chromatography with HiTrap Protein A HP column (Cytiva, USA).

The 1p2B5-Fc-LALA-PG antibody sequence was generated by introducing three point mutations (L234A, L235A, and P329G) using site-directed mutagenesis. 1p2B5-Fc-LALA-PG antibody was produced and purified according to the same procedures described above for 1p2B5-Fc.

### 2.6 SARS-CoV-2 neutralization assay

To evaluate the neutralizing activities of 1p2B5 and 1p2B5-Fc antibodies, a virus neutralization assay was performed. Vero E6 cells were seeded into 96-well culture plate. The following day, 100 TCID50 (50% tissue culture infectious dose) of authentic SARS-CoV-2 virus were mixed with serial dilutions of the antibodies in complete DMEM supplemented with 2% HI-FBS in a total volume of 100 μl for 1 hour at 37 °C. The samples were then transferred to a monolayer of Vero E6 cells followed by incubation at 37 °C and 5% CO_2_ for 96 h. The development of cytopathic effect (CPE) caused by the virus was assessed visually by light microscopy 4 days post-infection. The neutralizing concentration of each antibody was determined as the lowest antibody concentration that completely inhibited CPE in at least 2 of the 3 replicate wells.

### 2.7 Biolayer interferometry assay

The kinetics of 1p2B5 and the NTD of SARS-CoV-2 S protein binding were assessed using an Octet RED96 instrument (Sartorius, Germany) as described previously (14). Recombinant NTD of XBB.1 variant was biotinylated using an EZ-Link Sulfo-NHS-LC-Biotinylation Kit (Thermo Fisher Scientific, USA) and diluted in a kinetic buffer (PBS containing 0.02% Tween 20, 0.1% BSA and 0.05% sodium azide, pH 7.4) to a concentration of 10 μg/mL. Streptavidin biosensors were loaded with biotinylated NTD protein. The biosensors were then dipped into the wells of black 96-well plates (Greiner Bio-One, Austria) containing 400, 100, 25, 6.25 and 0 nM of 1p2.B5 antibody for 600 s. Dissociation was measured in the kinetic buffer without antibody for 700 s. The equilibrium dissociation constant (K_D_) was calculated by the Octet Data Analysis 10 software.

### 2.8 Membrane fusion inhibition assay

Vero E6 cells were seeded into a 96-well culture plate in complete DMEM medium at a density of 1x10^4^ cells per well. The following day, the cells were transduced with recombinant adenoviral vectors rAd5 encoding the S protein of XBB.1 variant and rAd5 encoding GFP at a dose of 2,000 virus particles of each vector per cell. Immediately after the addition of adenoviral vectors, the 1p2B5-Fc antibody or a control antibody (not binding to the S protein) was added to the wells at a concentration of 10 μg/mL. After incubation at 37 °C for 24 h, Vero E6 cells were imaged using an Olympus IX73 microscope. The formation of syncytia by the S protein expressing cells as a result of membrane fusion was assessed visually. Cells transduced with rAd5 encoding GFP alone was used as negative control for membrane fusion.

### 2.9 ADCC and ADCP assays

HEK293 target cells were seeded into a 96-well plate at a density of 2х10^4^ cells per well in complete DMEM medium. After 6 h of incubation at 37 °C and 5% CO_2_, the cells were transduced with the recombinant adenoviral vector rAd5 encoding the spike protein (Wuhan) at a MOI of 3 per cell and cultured in a 5% CO_2_ incubator for 48 h. Then, the medium was replaced with fresh medium containing serial dilutions of 1p2B5-Fc or 1p2B5-Fc-LALA-PG antibodies, ranging from 10 µg/mL to 0.001 µg/mL. After 1 h of incubation at 37 °C and 5% CO_2_, Jurkat-Lucia™ NFAT-CD16 or Jurkat-Lucia™ NFAT-CD32 effector reporter cells (Invivogen, USA) were added to the HEK293 cells at a concentration of 2x10^5^ cells per well, and the plate was placed in a 5% CO_2_ incubator for 16–18 h. Finally, the supernatants (30 µl) were transferred into a new 96-well white plate and mixed with 50 µl of QUANTI-Luc™ 4 Reagent working solution (Invivogen, USA) to assess luciferase activity. The luminescence was measured using a Synergy H4 microplate reader (Biotek, USA).

### 2.10 Ethics

The animal experiments were approved by the ethics committee of the Gamaleya Research Center (protocol #108, 13 Oct 2025).

### 2.11 Animal studies

All experiments were performed in accordance with the recommendations of the National Standard of the Russian Federation (GOST 33044–2014. Interstate standard. Principles of good laboratory practice).

The study of protective efficacy used Syrian hamsters weighing 60–80 g (Pushchino Nursery, health status SPF) and 6–7 weeks old hemizygous K18-ACE2-transgenic F1 mice (hACE2-transgenic mice) obtained from crossing transgenic males B6.Cg-Tg(K18-ACE2)2Prlmn/J (Jackson Laboratory, https://www.jax.org/strain/034860, health status SOPF) with non-transgenic females C57BL/6 Gamrc (Gamaleya Research Center, health status SPF). Animals had free access to water and food. Animals were housed in an ISOcage N system (Tecniplast, Buguggiate, Italy).

Syrian hamsters were infected intranasally with 10^5^-10^6^ TCID50 of SARS-CoV-2 virus (variants B.1.1.1, XBB.1.16, EG.5.1, XBB.1.9.1, JN.1 or KS.1). Then, the animals received 1p2B5-Fc antibody at a dose of 1 mg/kg in 100 μl of PBS intraperitoneally 1 h, 6 h or 24 h after virus inoculation. Control animals received 100 μl of PBS without antibody. Hamsters (n = 5 per group) were euthanized for lung tissue collection on the 4th day post challenge. For viral load determination, 10% lung homogenates were prepared in DMEM with 2% HI-FBS using MPbio FastPrep-24 instrument (MP Biomedicals, USA). The homogenates were centrifugated (12,000 g, 10 min), and serial dilutions of obtained supernatants were added to a monolayer of Vero E6 cells, followed by incubation for 120 h. The SARS-CoV-2 TCID50 titer virus was calculated by the Spearman–Kerber method, as described previously (14). The therapeutic activity of 1p2B5-Fc antibody was assessed by comparing the viral load in the lungs of antibody-treated animals and control animals using the Mann-Whitney test.

hACE2-transgenic mice were challenged with 10^5^ TCID50 of the SARS-CoV-2 B.1.1.1 (Wuhan D614G) variant, followed by 1p2B5-Fc or 1p2B5-Fc-LALA-PG antibody administration 1 h, 6 h or 24 h post infection. Mice survival and body weight changes were monitored for 5 animals from each group. Mice exhibiting weight loss exceeding 20% of their initial weight were humanely euthanized. To assess the viral load in lung tissue, 5 mice from each group were euthanized on day 4 post challenge, and lung samples were collected. The titer of infectious virus was determined as describe above.

## 3 Results

### 3.1 Single-domain antibody to NTD broadly neutralizes SARS-CoV-2 variants

The single-domain antibody 1p2B5 was identified as a promising neutralizing antibody targeting N-terminal domain (NTD) of SARS-CoV-2 S protein. It was isolated from a VHH phage display library obtained after serial immunization of Bactrian camel with immunogens containing the S protein of various virus sublineages (14). Clone 1p2B5 was obtained using phage library biopanning on the S protein of XBB.1 variant, followed by ELISA-based screening, and produced in *E. coli*.

**Figure 1:**
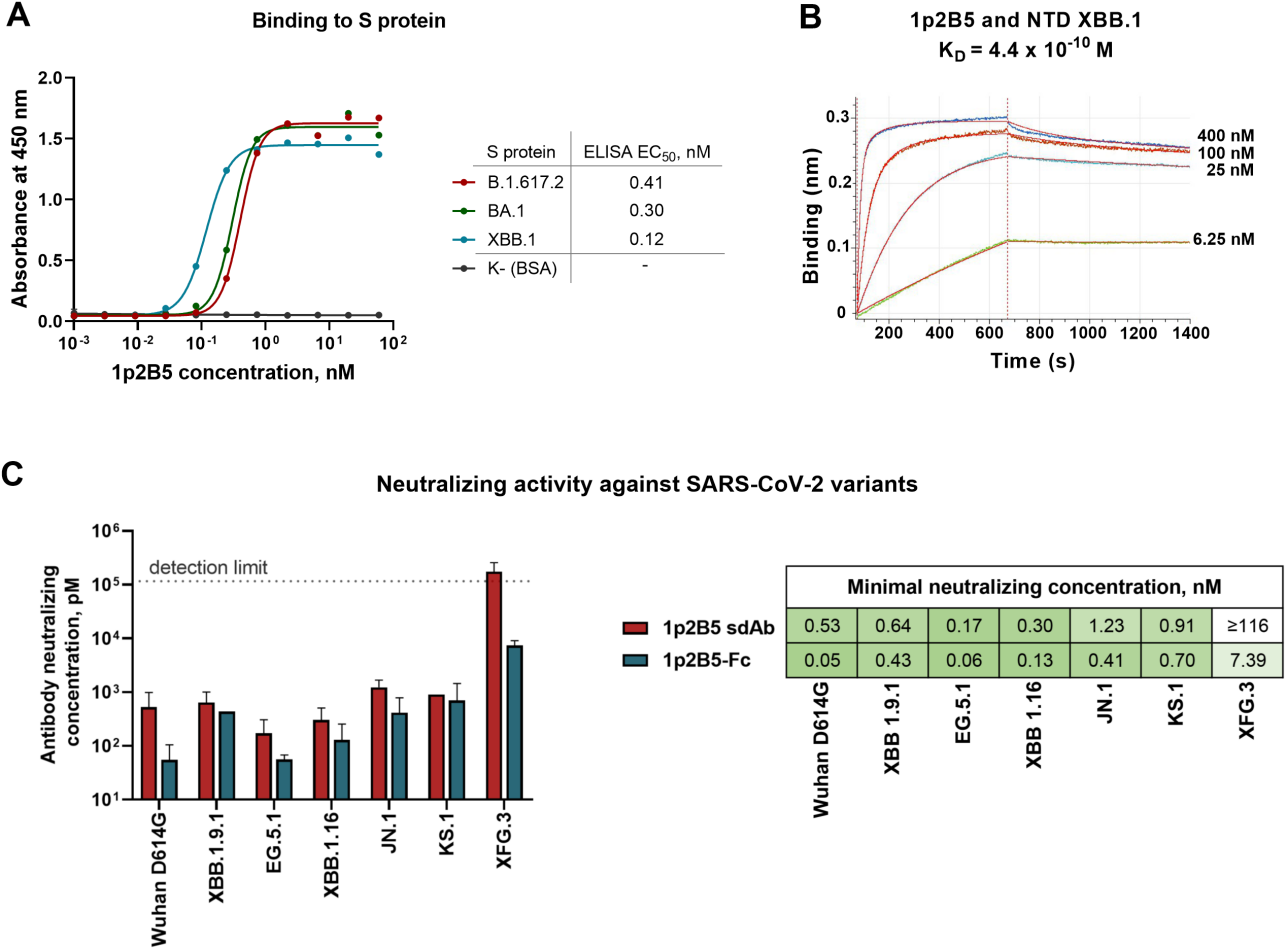
Binding and neutralizing activity of the 1p2B5 single-domain antibody. **(A)** Binding of 1p2B5 to the S protein of SARS-CoV-2 variants by ELISA. Dose-response curves and EC_50_ values are provided. **(B)** Bio-layer interferometry analysis of 1p2B5 binding to the NTD of XBB.1 variant. The equilibrium dissociation constant (K_D_) is indicated. **(C)** Neutralizing activity of 1p2B5 in single-domain and Fc-fused format against live SARS-CoV-2 variants. 100 TCID50 of SARS-CoV-2 virus (B.1.1.1 (Wuhan D614G), XBB.1.9.1, EG.5.1, XBB.1.16, JN.1, KS.1 or XFG.3 variants) were incubated (1 h, 37 °C) with serial two-fold dilutions of 1p2B5 sdAb or 1p2B5-Fc and added to a monolayer of VeroE6 cells for 96 h. Minimal neutralizing concentration was determined as the lowest concentration that completely inhibits the cytopathic effect of the virus.

To characterize the breadth of 1p2B5 activity, we first investigated its capacity to recognize the S protein of different SARS-CoV-2 sublineages. The analysis revealed that the antibody binds to the S protein of evolutionarily distant virus variants with high affinity. More specifically, 1p2B5 interacts with the S protein of Delta, Omicron B.1.1.529 and XBB.1 variants with ELISA EC_50_ values of 0.12-0.41 nM (**Figure 1A**). Kinetic analysis performed by bio-layer interferometry showed that dissociation constant (K_D_) of the 1p2B5 complex with NTD (XBB.1 variant) is 4.4*10^−10^ M (**Figure 1B**). Next, we assayed the *in vitro* neutralizing activity of 1p2B5 against diverse SARS-CoV-2 viruses circulating in 2020-2025, including the ancestral Wuhan D614G variant and the currently circulating XFG.3 virus. For this purpose, live SARS-CoV-2 viruses were mixed with serial dilutions of 1p2B5 antibody and added to Vero E6 cells. The minimal antibody concentration that completely inhibited the cytopathic effect (CPE) of each virus was then determined (**Figure 1C**). It was found that 1p2B5 potently neutralizes the Wuhan D614G, XBB.1.9.1, EG.5.1, XBB.1.6, JN.1, and KS.1 variants at concentrations of 0.17-1.23 nM. However, neutralizing activity against the XFG.3 variant was significantly reduced, as the antibody did not neutralize the virus variant at concentrations below 116 nM.

### 3.2 Fc-fused form of 1p2B5 has improved neutralizing activity

In the next step, we fused 1p2B5 sdAb with the Fc fragment of human IgG1, since this modification can enhance neutralizing capacity and provide broader activity (15–17). 1p2B5-Fc protein was produced through transient transfection of CHO-S cells and purified by protein A affinity chromatography. The binding of 1p2B5-Fc to the S protein was confirmed by ELISA (**Supplementary Figure 1**). *In vitro* neutralization assay using a panel of live SARS-CoV-2 viruses showed increased neutralizing activity of the Fc-fused form of 1p2B5 compared to the single-domain format (**Figure 1C**, P = 0.0156, Wilcoxon test, n=7). The 1p2B5-Fc antibody neutralizes the Wuhan D614G, XBB.1.9.1, EG.5.1, XBB.1.6, JN.1, and KS.1 viruses at concentrations of 0.05-0.7 nM, and also acquired the ability to potently inhibit the XFG.3 variant with a neutralizing concentration of 7.39 nM.

### 3.3 1p2B5-Fc protects animals from SARS-CoV-2 challenge

**Figure 2:**
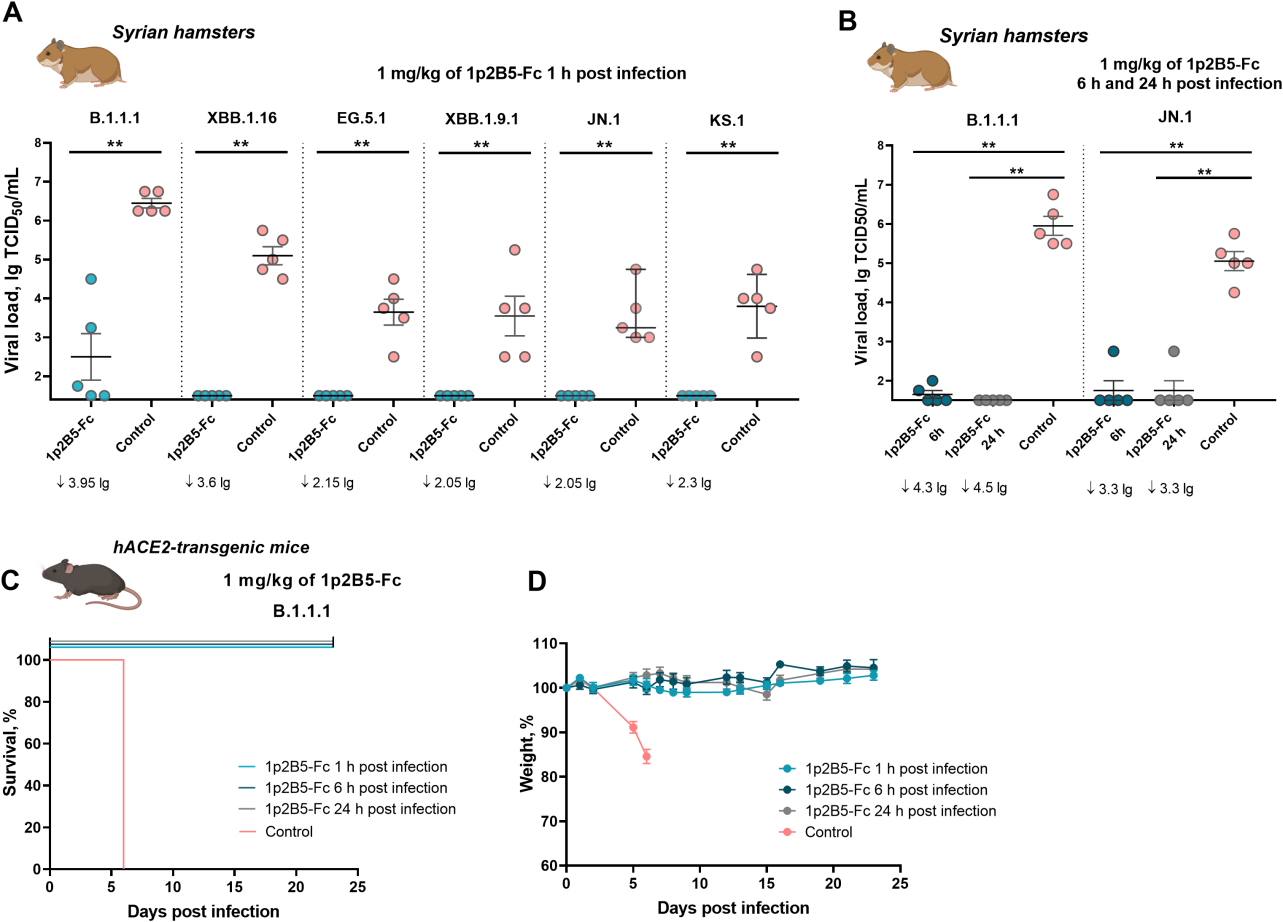
Protective efficacy of 1p2B5-Fc against SARS-CoV-2 challenge in animal models. **(A)** Syrian hamsters (5 animals per each antibody and control group) were challenged with SARS-CoV-2 variants (10^5^ TCID50 of B.1.1.1 (Wuhan D614G), XBB.1.16, EG.5.1, XBB.1.9.1, JN.1 viruses, 3x10^6^ TCID50 of KS.1 virus). 1 h post challenge hamsters were treated intraperitoneally with 1p2B5-Fc at a dose of 1 mg/kg or PBS (control groups). On day 4 post challenge, the infectious virus titer in lung tissue was assessed. **(B)** Syrian hamsters (5 animals per group) were challenged with SARS-CoV-2 B.1.1.1 (2x10^5^ TCID50) or JN.1 (10^6^ TCID50), followed by treatment with 1 mg/kg of 1p2B5-Fc 6 h or 24 h post challenge. Control groups received PBS instead of the antibody. Viral load in lung tissue was assessed on day 4 post infection. Significant differences between antibody and control groups were measured using a two-tailed Mann-Whitney test. **p<0.01. The values of viral load reduction in antibody group compared to control group are presented below the graphs. **(C, D)** K18-hACE2-transgenic mice (5 animals per group) were challenged with 10^5^ TCID50 of SARS-CoV-2 B.1.1.1 virus, followed by intraperitoneal injection of 1p2B5-Fc at a dose of 1 mg/kg 1h, 6 h or 24 h post challenge. **(C)** Graph shows survival (%) in antibody and control groups. **(D)** Graph shows weight dynamics in antibody and control groups.

The therapeutic efficacy of 1p2B5-Fc was studied in animal models. First, we examined the protective activity of 1p2B5-Fc in a Syrian hamster model of SARS-CoV-2 infection with different virus sublineages (Wuhan D614G (B.1.1.1), XBB.1.16, EG.5.1, XBB.1.9.1, JN.1, and KS.1). Syrian hamsters were challenged intranasally with SARS-CoV-2 viruses at a dose of 10^5^-3x10^6^ TCID50, depending on the virus variant. One hour post infection, animals received 1 mg/kg of 1p2B5-Fc intraperitoneally. On day 4 post challenge, the viral load in the lung tissue was assessed (**Figure 2A**). The results of the experiment showed that treatment with 1p2B5 significantly reduced infectious virus titer in the lungs in all SARS-CoV-2 variants studied. The reduction in viral load was more than 2 lg in the antibody groups compared to the control groups. The data obtained indicate the broad protective activity of the 1p2B5-Fc.

Next, we evaluated the therapeutic efficacy of the antibody when administrated at 6 h and 24 h post infection (**Figure 2B**). Syrian hamsters were intranasally inoculated with two evolutionarily distant variants of SARS-CoV-2, B.1.1.1 (2x10^5^ TCID50) and JN.1 (10^6^ TCID50), followed by intraperitoneal injection of 1p2B5-Fc at a dose of 1 mg/kg. Analysis of viral load in lung tissue on day 4 after challenge revealed a marked reduction in the titer of infectious virus (> 3 lg). Thus, 1p2B5-Fc also exhibits broad therapeutic efficacy when administered at later time points after SARS-CoV-2 challenge.

In addition, the therapeutic efficacy of 1p2B5-Fc was studied in a lethal model of SARS-CoV-2 infection of hACE2-transgenic mice. The mice were intranasally infected with 10^5^ TCID50 of the B.1.1.1 virus. Animals were treated with 1p2B5-Fc (1 mg/kg intraperitoneally) 1 h, 6 h, or 24 h post challenge. We observed that the antibody protected 100% of mice from lethal challenge with SARS-CoV-2 when administrated at any of the studied time points (**Figure 2C**). Analysis of weight dynamics showed that 1p2B5-Fc also protected animals from weight loss (**Figure 2D**).

### 3.4 The mechanism of neutralizing and protective activity of 1p2B5

**Figure 3:**
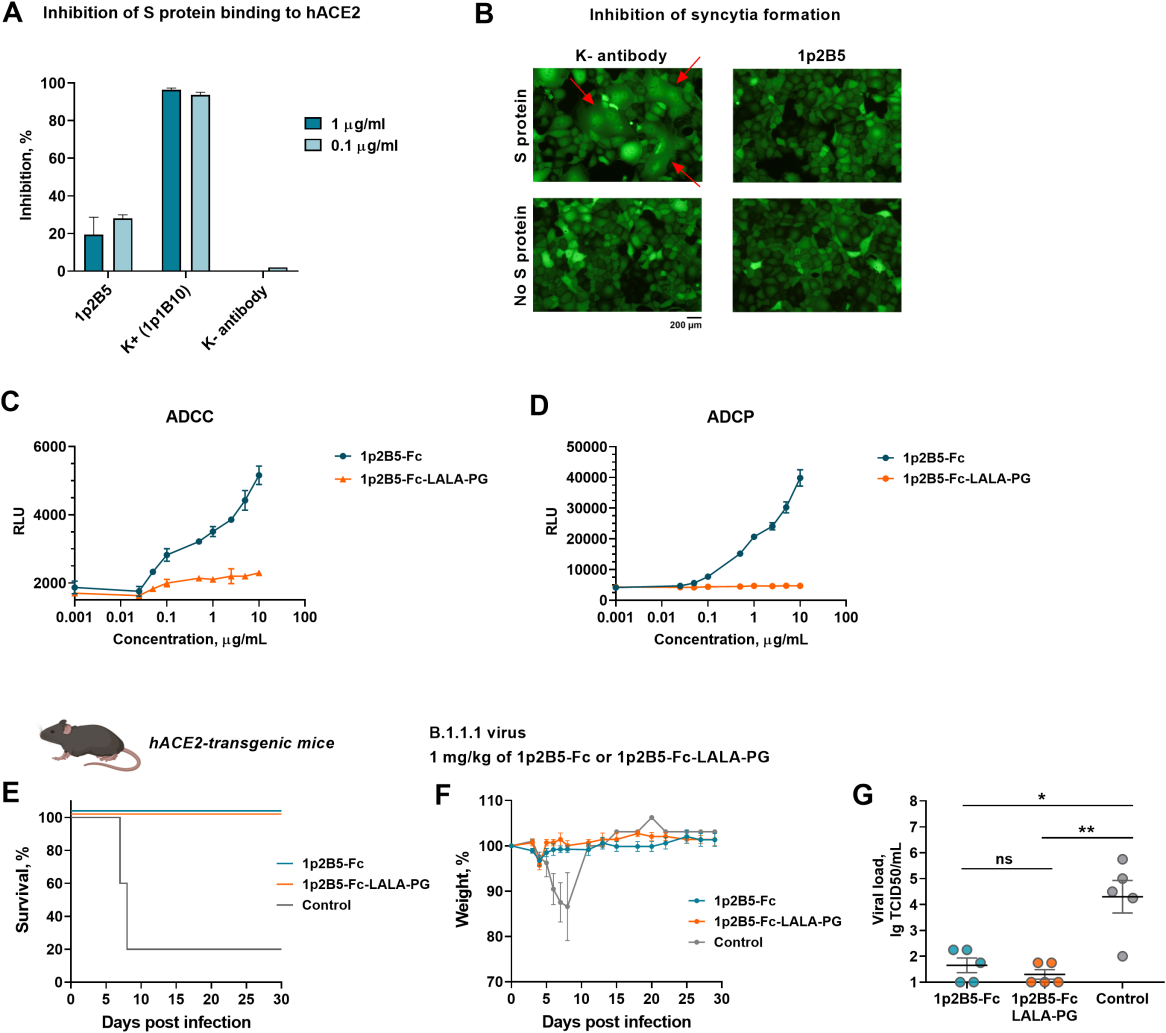
Mechanism of neutralizing and protective activity of the 1p2B5 antibody. **(A)** Inhibition of S protein binding to immobilized hACE2 receptor by 1p2B5, measured by competitive ELISA. A single-domain antibody (sdAb) 1p1B10 to S protein RBD was used as a positive control for inhibition, and a sdAb that does not recognize the S protein was used as a negative control. **(B)** Inhibition of syncytia formation by Vero E6 cells expressing S protein and GFP in the presence of 1p2B5. Vero E6 cells were transduced with recombinant adenoviral vectors rAd5 encoding S protein and rAd5 encoding GFP. Immediately after transduction, 1p2B5 or K- sdAb (which does not bind to the S protein) was added at a concentration of 10 μg/mL. The formation of syncytia was assessed 24 hours after transduction. The red arrows indicate the location of syncytia. Scale bar: 200 μm. **(C, D)** ADCC and ADCP assays measuring luciferase activation in Jurkat-Lucia™ NFAT-CD16 and Jurkat-Lucia NFAT-CD32 reporter cells after co-culturing with spike-expressing HEK293 cells in a presence of serial dilutions of 1p2B5-Fc or 1p2B5-Fc-LALA-PG. **(E–G)** K18-hACE2-transgenic mice (10 animals per group) were challenged with 10^5^ TCID50 of SARS-CoV-2 B.1.1.1 virus, followed by intraperitoneal injection of 1p2B5-Fc or 1p2B5-Fc-LALA-PG at a dose of 1 mg/kg 1 h post challenge. The control group received placebo (PBS). **(E)** Graph shows survival (%) in antibody-treated and control groups (n=5 per group). **(F)** Graph shows weight dynamics in antibody-treated and control groups (n=5 per group). **(G)** Five animals from each group were euthanized, and the infectious virus titer in lung tissue was assessed. Significant differences between groups were measured using a two-tailed Mann-Whitney test. *p <0.05, **p<0.01, ns, not statistically significant.

To identify the potential mechanism of SARS-CoV-2 neutralization by 1p2B5, we first assessed whether 1p2B5 could prevent the interaction of the S protein with ACE2 entry receptor. Recombinant hACE2 protein was immobilized on ELISA plates, then the S protein pre-incubated with 1p2B5 was added to the wells. We found that 1p2B5 provided only slight inhibition of S protein binding to ACE2, in contrast to the control blocking antibody 1p1B10 (**Figure 3A**). This result suggests that the mode of action of 1p2B5 unlikely mediated by interference with ACE2 receptor engagement.

Next, we investigated whether 1p2B5 could inhibit the subsequent step of SARS-CoV-2 entry, the membrane fusion process. For this purpose, Vero E6 cells were transduced with recombinant adenoviral vectors rAd5 encoding full-length S protein (XBB.1) and rAd5 encoding GFP. Expression of the S protein on the surface of Vero E6 cells leads to fusion of cell membranes and the formation of syncytia. Addition of 1p2B5-Fc immediately after transduction completely prevented the syncytia formation (**Figure 3B**). The result indicates that 1p2B5 could inhibit the virus-cell membrane fusion. At the same time, we assume that the antibody probably does not suppress S2′ cleavage (**Supplementary Figure 2**). The potential mechanism of 1p2B5 neutralizing activity could be related to premature S1 shedding, spike trimer disruption, or prevention of S protein interaction with an auxiliary receptor.

To assess the contribution of Fc-mediated effector functions to the therapeutic activity of 1p2B5-Fc, we introduced LALA-PG mutations (L234A, L235A, and P329G) to reduce the binding of 1p2B5-Fc to Fc gamma receptors. Then, we evaluated the ability of 1p2B5-Fc and its mutant form (1p2B5-Fc-LALA-PG) to induce antibody-dependent cellular cytotoxicity (ADCC) and antibody-dependent cellular phagocytosis (ADCP) using the Jurkat-Lucia™ NFAT-CD16 and Jurkat-Lucia NFAT-CD32 reporter cell lines. Analysis showed that 1p2B5-Fc dose-dependently activates both ADCC and ADCP, whereas the Fc-LALA-PG form does not induce any activity (**Figures 3C, D**). Next, we compared the protective activity of 1p2B5-Fc and 1p2B5-Fc-LALA-PG counterpart in a model of SARS-CoV-2 infection of hACE2 transgenic mice. The mice were inoculated with 10^5^ TCID50 of SARS-CoV-2 B.1.1.1 virus, and 1 hour post infection, the animals (n=10 per group) were treated with 1 mg/kg of 1p2B5-Fc or 1p2B5-Fc-LALA-PG intraperitoneally. Control animals received placebo instead of antibodies. It was found that both form of 1p2B5 protects 100% of hACE2 transgenic mice from lethal infection (**Figure 3E**). Animals from both antibody-treated groups showed no weight loss compared to the control group (**Figure 3F**). On day 4, five animals from each group were euthanized, and lungs were collected for viral load analysis. The infectious virus titer was significantly reduced in the lungs of animals that received 1p2B5-Fc or 1p2B5-Fc-LALA-PG (**Figure 3G**). No statistically significant differences in virus titers were found between the two antibody-treated groups. Thus, 1p2B5-Fc antibody could exert a protective efficacy *in vivo* due to its potent neutralizing activity without the involvement of Fc-mediated effector functions.

## 4 Discussion

The global spread of SARS-CoV-2 virus has caused a pandemic with a devastating impact on public health. The development of antiviral agents has become an essential component of the strategy to combat COVID-19. In the early years of the pandemic, a range of monoclonal neutralizing antibodies, such as sotrovimab, casirivimab and bamlanivimab, were developed and used for COVID-19 therapy. Over time, the emergence of new virus sublineages has led to a marked decrease in the efficacy of these antibodies, and their use was subsequently discontinued (2). Although much is known about the SARS-CoV-2 structure and variability, the development of therapeutic neutralizing antibodies that retain their activity against new virus subvariants is still a major challenge (1). Currently, there are no approved therapeutic monoclonal antibodies for COVID-19 treatment; however, pemivibart is authorized for prophylaxis (18). Most developed therapeutic antibodies recognize the RBD of the S glycoprotein, whereas targeting of non-RBD epitopes is also promising approach (4).

In this study, we describe the NTD-targeted broadly neutralizing single-domain antibody 1p2B5, previously isolated from a camel immune library (14). Although the NTD has accumulated numerous mutations, 1p2B5 demonstrated high affinity binding to the S protein of evolutionarily distant variants of SARS-CoV-2. Moreover, 1p2B5 inhibits multiple SARS-CoV-2 variants *in vitro*, including the earliest Wuhan D614G virus and more recent Omicron KS.1 subvariant, at low concentrations ranging from 0.17 to 1.23 nM. However, the currently circulating XFG.3 variant is inhibited only at a concentration of 116 nM, which may indicate that mutations have occurred in the 1p2B5 epitope region. Comparison of the S protein sequences of KS.1 and XFG.3 viruses revealed S31P, K182R and R190S substitutions in the NTD of XFG.3 variant (**Supplementary Figure 3**). Nevertheless, the Fc-fused bivalent form of 1p2B5 was able to potently neutralize the XFG.3 virus at a concentration of 7.39 nM, highlighting its broader neutralizing activity. We suggest that the improved activity of 1p2B5-Fc is related to increased valency and, as a consequence, to increased functional affinity. Enhanced neutralizing activity of the Fc-fused form of VHH compared to the single-domain format has also been demonstrated for other antiviral sdAbs (19, 20). Overall, the obtained results are encouraging for NTD-directed antibodies. Candidates for antibody therapy should demonstrate both broad activity and exceptional potency (21). However, anti-SARS-CoV-2 Abs rarely possess these properties concurrently, given the emergence of multiple genetically distinct virus sublineages (7, 10–12).

We next assessed the therapeutic efficacy of 1p2B5-Fc antibody using two animal models of SARS-CoV-2 infection. Studies on Syrian hamsters challenged with various SARS-CoV-2 variants (B.1.1.1, XBB.1.16, EG.5.1, XBB.1.9.1, JN.1, and KS.1) demonstrated a significant decrease in viral load in the lungs of animals treated with 1 mg/kg of 1p2B5-Fc 1 h after infection. Moreover, therapeutic administration of 1p2B5-Fc (1 mg/kg) at 6 and 24 h post-infection with B.1.1.1 and JN.1 also significantly reduced the titer of infectious virus in the lungs. We also evaluated *in vivo* protection of transgenic hACE2 mice using a lethal challenge model with Wuhan D614G (B.1.1.1). Administration of the antibody (1 mg/kg) at 1, 6 and 24 h post infection fully protected mice from lethal SARS-CoV-2 challenge. Our results underline the breadth of the antibody’s protective efficacy against diverse SARS-CoV-2 variants. Broad therapeutic efficacy is rarely reported for other published NTD-targeting antibodies (10, 11). For example, recent data showed that the 3711 Ab confers protection against wild-type SARS-CoV-2 when administered at a dose of 10 mg/kg one day post-challenge, but other variants of the virus have not been tested (10). Most NTD antibodies have been investigated only for prophylactic efficacy (5, 9, 11). The potent *in vivo* activity of 1p2B5-Fc at a low dose (1 mg/kg) emphasizes its strong potential for clinical development.

The promising broad neutralizing activity of 1p2B5-Fc required further elucidation of the mechanism of neutralization. SARS-CoV-2 neutralization can be achieved through several mechanisms acting either before or after the virus binding to ACE2 receptor. Therefore, we first investigated the capacity of 1p2B5 to inhibit the S protein interaction with ACE2 and found that 1p2B5 could not prevent the binding, but somewhat impeded the interaction. As reported, most NTD Abs also do not interfere with the S protein binding to ACE2, indicating other mechanisms of action (1, 7, 11). Some neutralizing Abs act at stages before receptor engagement. For example, they can induce conformational changes leading to destabilization of spike trimer or S1 shedding (4, 7, 9). Others NTD-targeted Abs act after ACE2 engagement by inhibiting ACE2-mediated S1 shedding or S2′ site cleavage (11–13). In a cell-cell fusion assay we demonstrated the ability of 1p2B5 to block the syncytia formation by S protein expressing Vero E6 cells. This finding suggests that 1p2B5 prevents the fusion of viral and host cell membranes and blocks SARS-CoV-2 entry into host cells. Further structural studies are required to shed light on the location and structure of the 1p2B5 epitope and to better understand the precise mechanisms of neutralization.

The *in vivo* therapeutic efficacy of an antibody could be mediated by its neutralizing activity, as well as by the involvement of antibody effector functions through the constant Fc region (11, 12, 22). To assess the contribution of Fc-mediated effector functions, we modified 1p2B5-Fc antibody to reduce binding with FcγRs by introducing LALA-PG point mutations. Then, the therapeutic efficacy of 1p2B5-Fc and 1p2B5-Fc-LALA-PG was compared in the model of SARS-CoV-2 infection of hACE2-transgenic mice. It should be noted that the capacity of human IgG1 to bind mouse FcγRs has been confirmed in previous studies (8). In addition, it has been shown that the introduction of LALA-PG modifications into human IgG1 also decreases the engagement of mouse Fc gamma receptors (23). We found that both 1p2B5-Fc and its LALA-PG mutant protect 100% of hACE2-transgenic mice from lethal infection and significantly reduce the infectious virus titer in the lungs. There were no statistically significant differences in the viral load between the two antibody groups. This result indicates that the observed protective efficacy of 1p2B5-Fc could be predominantly achieved through its potent neutralizing capacity.

Overall, we discovered NTD-specific single-domain antibody 1p2B5 with potent neutralizing activity against diverse SARS-CoV-2 variants. The Fc-fused form of the antibody neutralizes both the earliest and recently emerged virus variants *in vitro* and *in vivo*. Further investigation of 1p2B5 epitope could provide valuable insights about conserved neutralizing epitopes for prophylactic and therapeutic application.

## Data Availability

The raw data supporting the conclusions of this article will be made available by the authors, without undue reservation.
