# Supplementary material for: Broadly neutralizing single-domain antibody targeting the spike NTD displays therapeutic efficacy against diverse SARS-CoV-2 variants
Source: Front Immunol. 2026 Mar 23;17:1799333. doi: 10.3389/fimmu.2026.1799333 (PMC13050822; doi:10.3389/fimmu.2026.1799333)
Supplement: Supplementary file 1 [file DataSheet1.docx]

Supplementary Material

Supplementary Figures


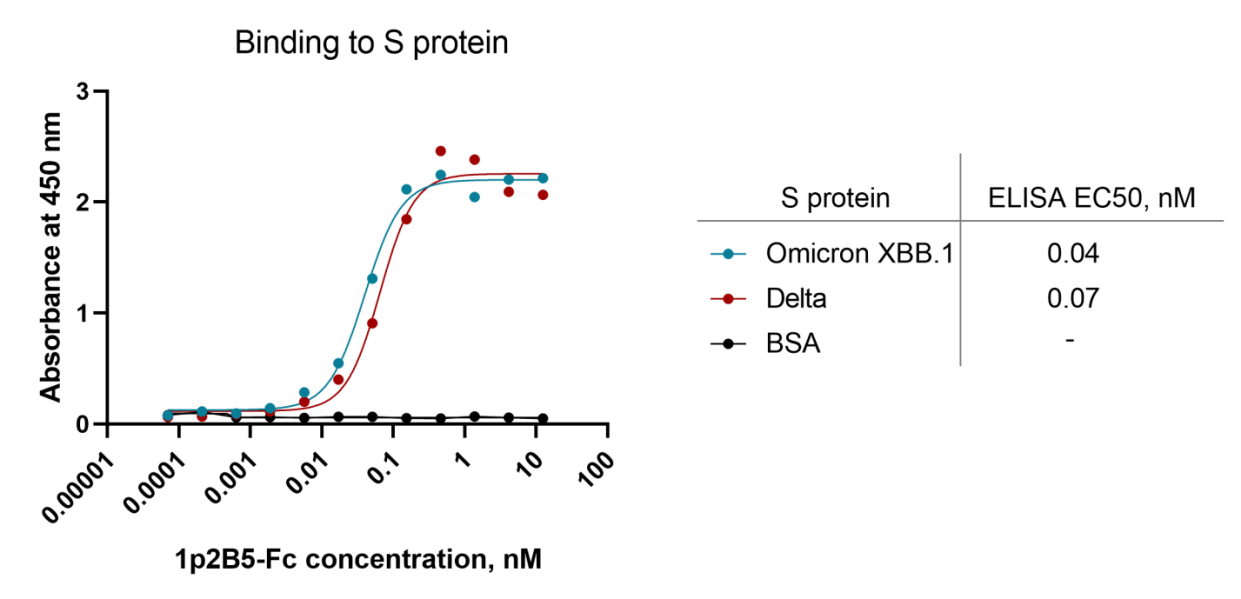


**Supplementary Figure 1.** 1p2B5-Fc binding to the S protein of SARS-CoV-2 variants. Dose-response curves and ELISA EC50 values are provided.

**
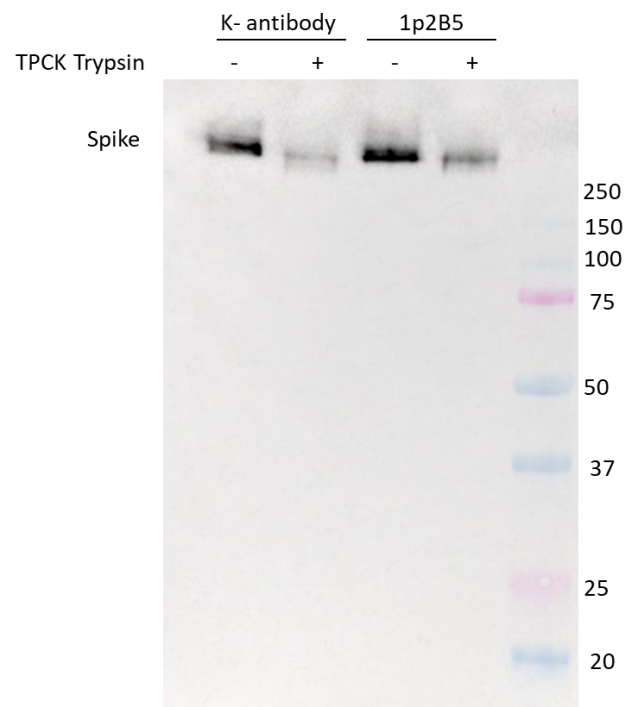
**

**Supplementary Figure 2.** S2′ cleavage assay. The S protein of XBB.1 (100 ng) was incubated with 1p2B5 or negative control antibody (120 ng) for 40 minutes at 37 °C. The samples were treated with TPCK Trypsin (20233, Thermo Scientific) (10 ng) for 15 min at 37 °C, and then were immediately placed on ice, and PMSF to a concentration of 1 mM was added. The capacity of 1p2B5 to block S2′ cleavage was examined by western blot analysis. The spike trimer (>250 kDa) was detected using anti-spike Abs (40150-R007, Sino Biological).


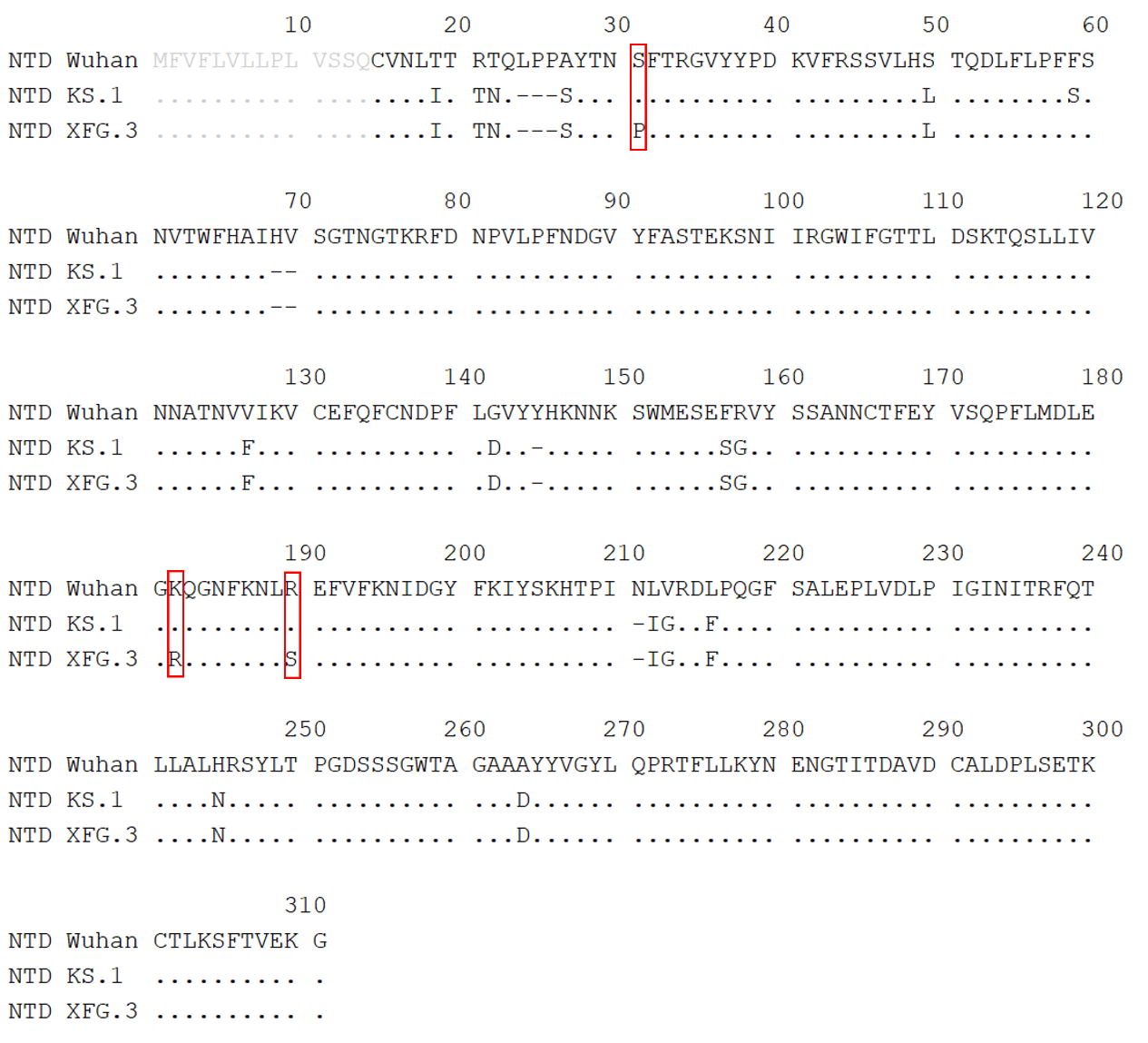


**Supplementary Figure 3.** The alignment of the S protein NTD sequences of Wuhan, KS.1 and XFG.3 variants. The S31P, K182R and R190S substitutions in the NTD of XFG.3 variant are highlighted with red rectangles.
